# Development and Performance of an Artificial Intelligence-Based Deep Learning Model Designed for Evaluating Dental Ergonomics

**DOI:** 10.3390/healthcare13182277

**Published:** 2025-09-11

**Authors:** Sanjeev B. Khanagar, Aram Alshehri, Farraj Albalawi, Sara Kalagi, Maryam A. Alghilan, Mohammed Awawdeh, Kiran Iyer

**Affiliations:** 1 Preventive Dental Science Department, College of Dentistry, King Saud Bin Abdulaziz University for Health Sciences, Riyadh 11426, Saudi Arabia; 2 King Abdullah International Medical Research Center, Ministry of National Guard Health Affairs, Riyadh 11481, Saudi Arabia; 3 Restorative and Prosthetic Dental Sciences Department, College of Dentistry, King Saud bin Abdulaziz University for Health Sciences, Riyadh 11426, Saudi Arabia

**Keywords:** artificial intelligence, automated, assessment, deep learning, dental posture, ergonomics, evaluation, musculoskeletal disorders, prevention, work postures

## Abstract

**Background/Objectives**: Dental professionals engage in a variety of dental procedures within a confined workspace that is often challenging to access and navigate. This environment frequently results in static, asymmetrical, and inappropriate postures, which can lead to muscular imbalances and cause pain or damage to the musculoskeletal system. Such issues can adversely affect the dental workforce, resulting in increased absenteeism, reduced productivity, disability, and premature retirement from the profession. Therefore, the objective of this study was to develop and evaluate the performance of an Artificial Intelligence (AI)-based deep learning model designed to assess dental ergonomics. **Methods**: An AI-based Dental Ergonomic Posture Assessment Model [SBK-DentErgo] was developed through the strategic integration of YOLOv11 and MediaPipe. Model training and validation were conducted using 500 photographs of dental professionals performing procedures on patients, captured from both frontal and sagittal planes. In the initial phase of the study, two calibrated evaluators assessed 50 photographs, demonstrating excellent agreement. In the subsequent phase, five dental specialists, along with the AI model, evaluated the same set of photographs, and the results were recorded. **Results**: AI-based model demonstrated excellent agreement with that of calibrated evaluators (Kappa = 0.922, *p* = 0.000). The reliability of AI-based scores was also consistent (ICC = 1.000, *p* = 0.000). Human evaluation of ergonomic posture exhibited very low sensitivity (20.5%) compared to AI, which showed very high sensitivity (97%). The specificity of human evaluation was also extremely low (9.1%) in contrast to AI (85.7%). The AI model (AUC = 0.917, 95% CI 0.762–1.000) could serve as the ‘gold standard’ in evaluating dental operator ergonomics. **Conclusions**: This AI model demonstrated exceptional performance in evaluating the working postures of dental professionals, surpassing experienced specialists in both sensitivity and specificity. The model provides real-time feedback, enabling dentists to conduct self-assessments and correct their posture immediately, thereby preventing postural issues.

## 1. Introduction

Dental professionals deliver dental care to their patients by performing a wide range of dental procedures in a compact workspace that is challenging to access and navigate. This environment often leads to static, asymmetrical, and inappropriate postures [1]. Frequent occurrences of inappropriate postures, including repetitive and prolonged motions such as sideways tilting of the head, neck, and torso, as well as stationary postures like standing or sitting with a forward bend, are very common in dental clinics [1,2,3].

As the body’s posture deviates further from a neutral position, the muscles responsible for bending or rotating toward the preferred side become stronger, while the opposing muscles weaken and lengthen, resulting in muscular imbalances. Muscle pain or damage to the musculoskeletal system can further hinder daily activities, leading to musculoskeletal disorders (MSDs) [4]. When the work environment and job performance significantly contribute to the development of chronic, progressive musculoskeletal conditions involving ligaments, spinal discs, muscles, cartilage, nerves, joints, and tendons, these conditions are classified as work-related musculoskeletal disorders (WMSDs) [5].

Inappropriate body positions while working can lead to pain, muscle spasms, joint rigidity, shivering, and disruptions in the daily lives of dentists. These issues may result in fatigue, tingling, pain, and numbness in the wrists, shoulders, lower back, and neck [6,7]. Common hand and wrist conditions include tendinitis/tenosynovitis, De Quervain’s disease, trigger finger, carpal tunnel syndrome, and Guyon’s syndrome [8]. These musculoskeletal disorders (MSDs) are primarily caused by repetitive hand motions, incorrect hand positions, strong gripping, mechanical stress on the palm, and exposure to vibration [4,9].

Under normal circumstances, rest periods facilitate the repair of damaged tissues. However, due to insufficient rest, the rate of damage in dentistry exceeds the rate of repair, potentially leading to muscle necrosis [1]. A systematic review reported that 64% to 93% of dental professionals worldwide experience work-related musculoskeletal disorders (MSDs) [10]. The most prevalent areas affected by these disorders among dental professionals were identified as the lower back (29% to 94.6%), shoulder (25% to 92.7%), and neck (26% to 92%) [11].

These musculoskeletal disorders (MSDs) can negatively impact the dental workforce, resulting in increased sick leave, decreased productivity, disability, and early retirement from the profession [8]. Considering the ergonomic risks in dentistry, it is crucial to implement effective ergonomic interventions to prevent or address these issues. Adhering to the precise rules and precautions outlined in ergonomic guidelines is essential to avoid the onset of work-related musculoskeletal disorders (WMSDs) among dental professionals [12,13].

An important first step in addressing risks and enhancing worker protection is identifying ergonomic risk factors in the workplace. However, data on the ergonomic postural assessment of dental professionals from previous research are extremely limited. The most commonly used methods for assessing the working postures of dental professionals include the Rapid Upper Limb Assessment (RULA) [14], the Rapid Entire-Body Assessment (REBA) [15], the Standardized Photometric Assessment Method (SPAM) [16,17], the Posture Assessment Criteria (PAC) [18], the Posture Assessment Instrument (PAI) [19], and the Modified Dental Operator Posture Assessment Instrument (M-DOPAI) [20].

Ergonomic evaluations of working postures in the dental profession typically utilize observational methods and are generally conducted by human experts. However, human error is inevitable in manual assessments due to variations in intra-rater and inter-rater reliability, which ultimately compromise the reproducibility of these evaluation measures. Hence, considering the recent breakthrough in technology, Artificial Intelligence (AI) has emerged because of recent scientific and technological advancements, and it has been extensively used in the health sector for a variety of tasks. In the field of dentistry, AI has been widely used to perform a variety of tasks, including diagnosis, clinical decision-making, and treatment prognosis prediction [21,22]. According to reports, AI-based automated systems perform exceptionally well. In certain studies, it was discovered that these systems could even surpass dental specialists in terms of accuracy and performance, imitating the precision and accuracy of skilled professionals [21].

Hence, with this background, this study intends to develop a novel AI-based model designed to evaluate the ergonomic sitting positions adopted by dental professionals while performing dental procedures. The AI model will provide real-time feedback, allowing dentists to correct their posture immediately and thereby prevent postural problems. Ultimately, this approach can positively impact dental professionals by improving their ergonomic habits and reducing the risk of musculoskeletal disorders associated with poor posture.

Hence, the aim of this study was to develop and assess the performance of an AI-based deep learning model designed to evaluate the ergonomic sitting positions adopted by dental professionals while performing dental procedures. The objectives were to develop an AI model that can detect and analyze key body landmarks using a combination of YOLOv11 and MediaPipe. To classify working postures into acceptable, compromised, or harmful using validated ergonomic criteria. To compare the performance of AI-based posture classification with human evaluators in terms of sensitivity, specificity, and reliability. We hypothesized that the AI-based model would achieve higher sensitivity and specificity in identifying compromised and harmful postures compared to human evaluators.

## 2. Materials and Methods

### 2.1. Study Design

This is a comparative study. Before initiating the research, ethical clearance was obtained from the Institutional Review Board at the King Abdullah International Medical Research Center in Riyadh, Saudi Arabia (KAIMRC) under protocol number NRR24/053/12. This study was conducted from December 2024 to June 2025.

### 2.2. Data Collection

We recruited five dental faculty members with no history of musculoskeletal disorders who were willing to participate in the study. A total of 500 photographs were taken of the dentists performing dental procedures on patients in the dental clinics of the College of Dentistry at King Saud bin Abdulaziz University for Health Sciences in Riyadh, Saudi Arabia. Written informed consent was obtained from the dentists and the patients after explaining the purpose of the photographs. Photographs and videos were recorded using two iPhone 15 (Apple Inc., Cupertino, CA, USA) mobile phones, each equipped with a dual-camera system on the rear, including a 48MP Main lens with a f/1.6 aperture and a 12MP Ultra-Wide lens with a f/2.4 aperture. Both devices were mounted on a leveled tripod: one positioned on the left (sagittal plane) at a 90-degree angle to capture the side profile, and the other facing the operator (frontal plane) to record the front view. The cameras were placed 1.5 m away from the dental chair, at a height of 1 m, ensuring they captured the participants’ working area, which facilitated the collection of data required for the study. These methods were considered after reviewing previous articles published by Partido, B.B. [20], Pîrvu, C. et al. [23], and Zúniga, I.A.C. et al. [24]. We utilized iPhones for capturing the photographs; however, we did not apply specific lens distortion corrections, and we accounted for environmental variability. To simulate various office lighting conditions, we incorporated color augmentations during the training phase. This approach enhanced the model’s ability to generalize across different levels of brightness, contrast, and color temperature. We refrained from performing distortion correction to preserve the natural capture characteristics of the devices.

### 2.3. Model Development

The Dental Ergonomic Posture Assessment Model [SBK-DentErgo] was developed by strategically integrating YOLOv11 (You Only Look Once Version 11) and MediaPipe to achieve accurate and robust human pose estimation. YOLOv11, a state-of-the-art object detection and pose estimation model, was selected for its exceptional ability to accurately localize multiple anatomical landmarks simultaneously, ensuring effective real-time inference suitable for clinical applications [25,26]. It provides reliable predictions for critical body landmarks aligned with the COCO (Common Objects in Context) dataset standard, including the shoulders, elbows, wrists, hips, knees, and ankles, which are essential for evaluating posture-related ergonomics.

**Figure 1:**
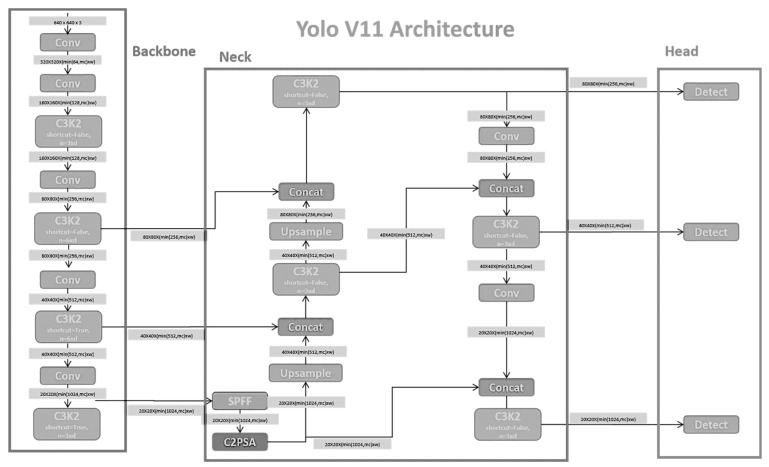
YOLOv11 Architecture.

However, YOLOv11 alone lacks the necessary granularity for detailed hand and finger landmarks, which are essential for accurately assessing wrist posture deviations commonly encountered by dental professionals. To address this limitation, MediaPipe’s pose detection framework was integrated to enhance YOLOv11’s predictions. MediaPipe provides a comprehensive set of 33 keypoints, including high-resolution landmarks for hands and fingertips, enabling precise wrist angle analyses that are crucial for ergonomic assessments. Details of the YOLOv11 architecture are provided in Figure 1. Additional details are provided in Abbreviations.

**Figure 2:**
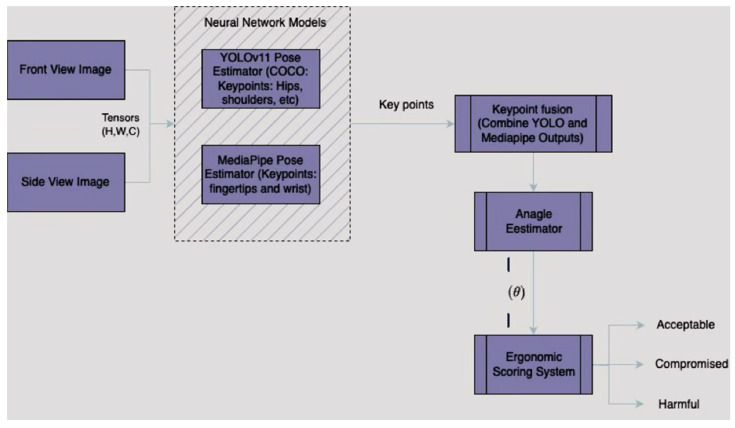
Combined YOLOv11 and MediaPipe outputs.

The combined outputs of YOLOv11 and MediaPipe were processed through a customized post-processing pipeline specifically designed for ergonomic posture evaluation. This pipeline calculates angles between anatomically relevant joint triplets using vector-based geometric analysis. Specifically, torso inclination is derived from the hip, shoulder, and knee landmarks; neck flexion is assessed using the hip, shoulder, and ear; upper-arm angles are determined from the shoulder, elbow, and wrist; and wrist deviations are calculated from the coordinates of the wrist, index fingertip, and pinky fingertip. Each calculated angle is categorized into ergonomic risk zones—acceptable, compromised, or harmful—based on established ergonomic standards adapted from the Modified Dental Operator Posture Assessment Criteria (M-DOPAC), which was developed from the Posture Assessment Instrument by Branson et al. [27] (Figure 2).

This M-DOPAC consists of ten components related to posture. The hips and legs are combined into one component, while the trunk is divided into two components. Similarly, the head and neck, upper arms, and wrists each consist of two components. Additionally, the position of the shoulders includes two components. In this assessment, we maintained the wrist position, which was considered in the Posture Assessment criteria [18].

**Figure 3:**
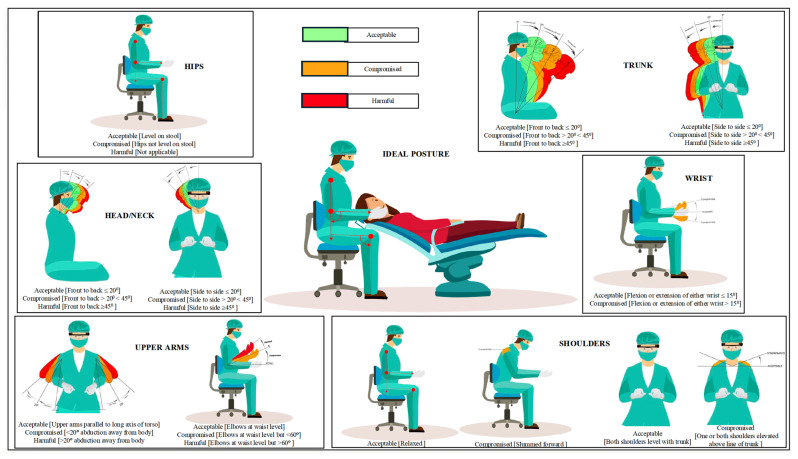
Modified-Dental Operator Posture Assessment Criteria (M-DOPAC).

We incorporated an additional component into the pre-validated Posture Assessment Instrument developed by Branson et al. [27]. The authors reported on the face validity of the scale, which was evaluated by subject experts. They found that most variations (80%) in scores were attributed to postural differences rather than inter-rater variability, thereby confirming its validity as a tool for assessing posture. The M-DOPAC is utilized to categorize each component as acceptable, compromised, or harmful, indicating the degree of deviation from Nield-Gehrig’s ideal posture [28]. Ideal postures are depicted in both side profiles and front views (Figure 3).

Each component is assigned a score in one of three categories: acceptable (1 point), compromised (2 points), or harmful (3 points). Only six components fall into the harmful category. Consequently, the scores range from 10 to 26, with the most ideal postures receiving a score of 10 points. Scores between 11 and 20 are considered compromised, while scores of 21 and above are classified as harmful. (Table 1).

**Figure 4:**
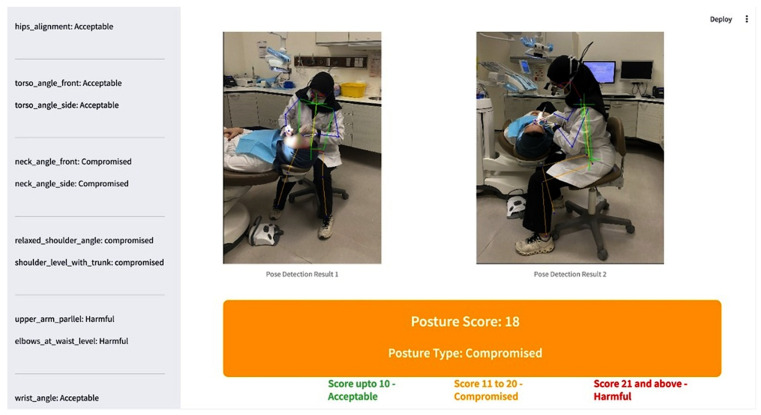
SBK-DentErgo Model—based Ergonomic Evaluation.

The final implementation utilized Streamlit to develop an interactive user interface that effectively presents ergonomic scores, visually highlighting risk areas through color-coded feedback (Figure 4).

This integration not only enhances usability in dental clinical settings but also delivers immediate actionable feedback, facilitating proactive ergonomics management and minimizing the risk of work-related musculoskeletal disorders.

### 2.4. Labelling of Data

The labeling of data for this model was automated based on anatomical keypoints, thereby eliminating the need for manual pixel-level annotations. Semantic labels were derived using angular thresholds grounded in ergonomic research literature (e.g., M-DOPAC). Specific criteria include categorizing a neck angle greater than 45° as harmful, upper arm elevation exceeding 20° as either compromised or harmful, and wrist deviation beyond 15° as compromised. Additionally, keypoints were generated using MediaPipe on a custom dataset specifically collected for task-oriented ergonomic assessments.

### 2.5. Data Processing

Data preprocessing involved several critical steps to ensure accuracy and consistency. The raw image data collected from dental practice sessions were resized and normalized to meet the input specifications required by YOLOv11 and MediaPipe. This normalization step ensured uniformity in the input data, reducing variability and enhancing the reliability of pose estimation results.

To address data quality issues, a preprocessing method utilizing a temporal window of 15 frames (0.5 s) was implemented. Missing or inaccurately detected keypoints from YOLOv11 and MediaPipe were identified within this window and replaced with the mean coordinates calculated from adjacent frames, thereby minimizing noise and enhancing data consistency.

### 2.6. Training and Testing

The combined dataset was divided into training, validation, and testing subsets, following an 80:10:10 ratio. Data augmentation techniques specific to pose estimation, such as random horizontal flipping, scaling, and rotation, were applied to the training dataset to enhance the model’s robustness and generalization. The training subset was utilized to train the ergonomic posture estimation model, which employed supervised learning algorithms optimized through backpropagation. Model parameters and hyperparameters were systematically tuned using cross-validation to enhance predictive performance and minimize overfitting. The testing subset was reserved to independently evaluate the generalization capabilities and reliability of the trained model.

### 2.7. AI-Based SBK-DentErgo Model Performance Evaluation

The evaluation of ergonomic postures for the test sets was conducted by two dental public health specialists with extensive academic experience and research backgrounds. Both specialists were calibrated on the assessment criteria and demonstrated substantial agreement, with a 92% level of agreement measured by Cohen’s kappa. The test sets were assessed by these two specialists, and their outcomes were established as true scores, serving as the ground truth.

Five dental specialists with good academic and research backgrounds were approached to evaluate photographs for ergonomic postures. The same set of photographs was assessed by the faculty after a two-week interval to determine reliability. For testing the models, the same set of photographs was manually uploaded to the AI model to categorize the working postures and record the performance of the AI model in comparison with the five dental specialists. All results were recorded in MS Excel and subjected to statistical analysis.

### 2.8. Statistical Analysis and Evaluation Criteria

All relevant data were initially recorded in Microsoft Excel (Microsoft Corp., New York, NY, USA) and later transferred to the Statistical Package for the Social Sciences (SPSS) software, Version 25 (IBM Corporation, Armonk, NY, USA), for analysis. As the first step, Cohen’s Kappa was used to assess the inter-rater variability among calibrated evaluators, the AI-based model, and human evaluators. Intra-class correlation (ICC) was used to analyze the intra-rater variability after a two-week gap for both AI and human evaluators.

Following this, model performance was rigorously evaluated using standard classification metrics, including sensitivity, specificity, positive predictive value (PPV), and negative predictive value (NPV), along with confidence intervals. The confusion matrix enabled comprehensive visualization of true positives, true negatives, false positives, and false negatives.

Sensitivity provided the model’s ability to correctly identify instances of harmful or compromised postures, while specificity quantified the model’s ability to identify acceptable postures correctly. Predictive values provided insight into the proportion of correctly classified harmful or compromised postures among all predicted harmful or compromised instances.

A graphical representation of the Receiver Operating Characteristic (ROC) curve, along with the Area Under the Curve (AUC), was generated for both AI-based diagnosis and human evaluation to facilitate a comparison of sensitivity and specificity-based output.

## 3. Results

Cohen’s kappa analysis was carried out to assess inter-rater agreement among principal investigators based on their evaluation of ergonomic scores for fifty sets of operator postures. A high level of agreement was observed in the scoring patterns among the principal calibrated evaluators (Kappa = 0.924, *p* = 0.000) (Table 2).

Cohen’s Kappa analysis was further applied to assess two types of inter-rater reliability: first, between the AI-based model and calibrated evaluators, and, second, among five independent evaluators who evaluated 10 random sets of operator postures. These 10 sets were part of a total of 50 sets analyzed by the AI.

Contrastingly, Cohen’s kappa analysis revealed that the inter-rater reliability between the AI-based model and calibrated evaluators was close to perfect agreement (Kappa = 0.922, *p* = 0.000). The agreement between the scores generated by the AI-based model and those of the human evaluators for ergonomic postures indicated that three of the five human evaluators tended to score more negatively, presuming that the operator was less compromised in their posture. Only one evaluator (the fifth) demonstrated moderate agreement (Kappa = 0.429, *p* = 0.050) with the scores generated by the AI model (Table 2).

Intra-evaluator scores were analyzed after a two-week interval to assess the reliability of scoring ergonomic postures by human evaluators and an AI-based model. Similar to the observations made using Cohen’s Kappa, the intra-class correlation analysis revealed nil to poor reliability in the scoring patterns of the four evaluators; only one evaluator (the fifth) demonstrated moderate reliability (average measures ICC = 0.597, *p* = 0.106). In contrast, the AI-based scores were consistent and comparable when assessed at two-week intervals (average measures ICC = 1.000, *p* = 0.000) (Table 2).

As the second step, AI-based scores and human evaluation scores were subjected to sensitivity and specificity analysis through cross-tabulation. AI scores were compared against those of calibrated evaluators, while the scores from random human evaluators were also compared to the AI scores. This analysis aimed to evaluate the performance differences between human evaluators and AI. Initially, the scores were categorized into positive and negative assessments. The human evaluation, which served as a diagnostic judgment for the ergonomic posture of the operator, demonstrated very low sensitivity (20.5%) compared to the significantly higher sensitivity (97%) of the AI-based model in detecting compromised posture, considering the standard cut-off for typical diagnoses to be 80% [29]. Similarly, the specificity of human evaluation was also very low (9.1%) compared to the high specificity (85.7%) of the AI in identifying appropriate posture. The human evaluation was not within the normal acceptable range required for diagnostic tests, whereas AI consistently provides reliable results for accurate diagnosis (Table 3).

The positive predictive value (PPV) for AI-based model (B = 0.977, 95% CI 0.902–0.999) and human evaluation (B = 0.444, 95% CI 0.234–0.670), as well as the negative predictive value (NPV) for AI-based model (B = 0.857, 95% CI 0.506–0.991) and human evaluation (B = 0.031, 95% CI 0.002–0.130), clearly delineate the efficiency of AI-based model in accurately categorizing postures. In contrast, human evaluation demonstrates a consistent tendency for flawed assessments in diagnosing operator posture. The Odds Ratio for appropriate posture diagnosis, as indicated in the risk estimate cohort (Actual 1), shows that AI (OR = 6.837, CI 1.113–41.995) significantly outperforms human evaluation (OR = 0.226, 95% CI 0.118–0.430) in determining correct posture diagnosis (Table 4 and Table 5).

**Figure 5:**
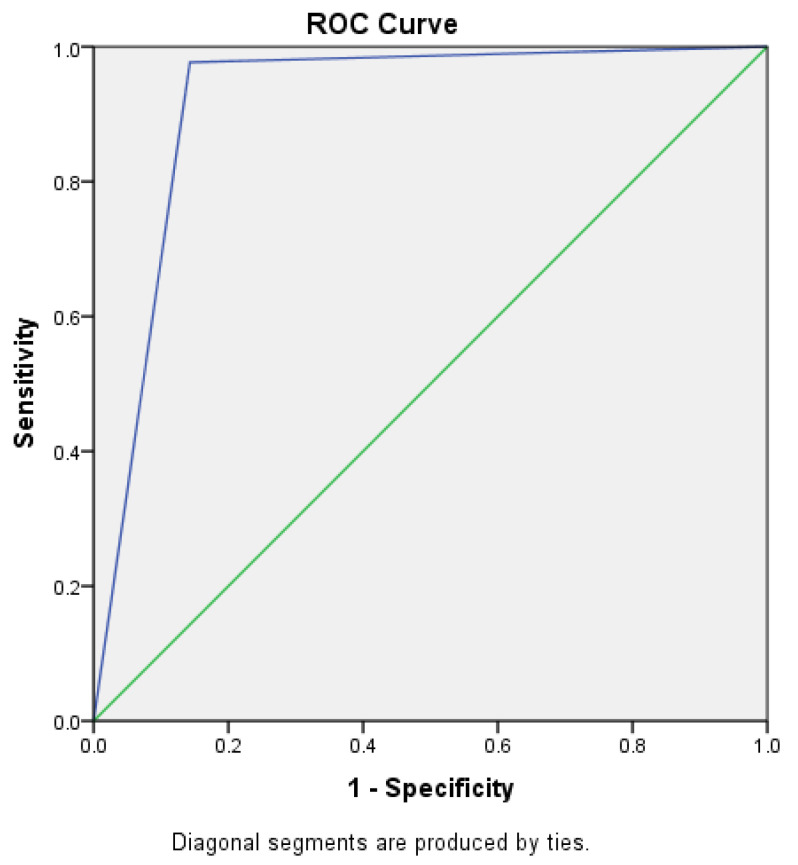
Receiver Operating Curve (ROC) for AI-based Diagnosis of Dental Operator Ergonomics. Green line—diagonal line of comparison. Blue line—true positive against false positive for human evaluation.

**Figure 6:**
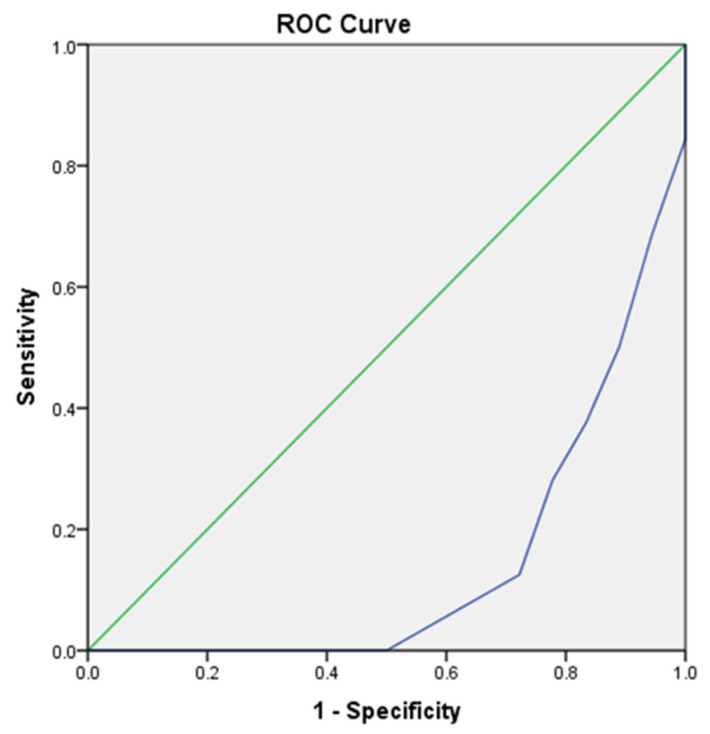
Receiver Operating Curve (ROC) for human evaluation of Dental Operator Ergonomics. Green line—diagonal line of comparison. Blue line—true positive against false positive for human evaluation.

The Receiver Operating Characteristic (ROC) curve, derived from the dichotomized posture assessment (where positive indicates compromised posture and negative indicates acceptable posture), demonstrates the effectiveness of AI-based model diagnosis compared to human evaluation. The AI-based model achieved an Area Under the Curve (AUC) of 0.917 (95% Confidence Interval [CI]: 0.762–1.000), indicating a high true positive rate. In contrast, human evaluation showed a significantly lower AUC of 0.143 (95% CI: 0.027–0.259), reflecting a high rate of false positive diagnoses (Table 6 and Figure 5 and Figure 6).

## 4. Discussion

The existing literature confirms the significant prevalence of musculoskeletal disorders (MSDs) among dentists [30,31]. This elevated percentage is partially attributed to the adoption of unfavorable working postures, insufficient breaks, and prolonged hours spent in static positions [32]. Static posture during work is identified as the primary risk factor for MSDs [33]. Furthermore, the symptoms of MSDs tend to worsen with the number of years in practice [34,35].

In this study, two views of positions were analyzed, one frontal and the other sagittal, to capture the clinician’s body posture during procedures. The images were captured and analyzed using MDOPA, a tool specifically designed to assess the working postures of dental operators, focusing on the entire body with particular emphasis on the upper body, as dental procedures are performed in a seated position. Utilizing photography for ergonomic evaluations has demonstrated its effectiveness as a viable and practical approach to enhance operators’ self-awareness and posture [36,37].

In this study, we developed an AI-based SBK-DentErgo model that serves as an automated observational technique specifically designed to evaluate the working postures adopted by dental practitioners. The effective use of deep learning in the field of posture recognition has significantly improved the accuracy and generalization capabilities of two-dimensional (2D) posture recognition, where datasets are crucial to the system [38]. In the present study, a substantial number of datasets were utilized for training and testing the model. Three-dimensional (3D) posture recognition was not implemented due to challenges associated with 3D posture labeling and high costs [38,39]. However, it is important to note that 2D poses do not allow for any rotation in 3D space. Consequently, we had to eliminate two 3D aspects, rotation between the planes, one for the neck and another for the shoulders, from the MDOPA.

One of the primary challenges in posture recognition is human body occlusion, especially in the context of multi-person posture recognition, which is often encountered in natural environments. Human body occlusion includes not only the obstruction caused by the body itself but also the interference from other objects and individuals that may obscure visibility [40,41]. Occlusion, where parts of the body are blocked either by the dentist’s own limbs or by instruments and equipment, presents a common challenge in computer vision-based posture analysis. To minimize the impact of occlusion in this study, a dual-camera setup was employed—capturing images from both the frontal and sagittal views simultaneously. This ensured that if a particular anatomical landmark was not visible in one plane due to self-occlusion or obstruction, it could often still be detected in the other. Furthermore, the use of advanced pose estimation models—YOLOv11 and MediaPipe—helped mitigate partial occlusions by leveraging context from visible keypoints to infer the positions of hidden or partially visible joints. While complete occlusion of certain landmarks can still limit accuracy, the combined use of multiple views and high-resolution models significantly improved the robustness of pose estimation in clinical scenarios.

In the current research, we have developed a model that employs cutting-edge technology and demonstrates exceptional performance. The human evaluation, which served as a diagnostic assessment of the operator’s ergonomic posture, demonstrated a very low sensitivity of 20.5% compared to the significantly higher sensitivity of 97% exhibited by the AI-based model in detecting compromised posture. Similarly, the specificity of the human evaluation was also very low at 9.1%, in contrast to the high specificity of 85.7% achieved by the AI in identifying appropriate posture. The human evaluation was not within the acceptable range required for diagnostic tests [29], whereas the AI consistently provides reliable results for accurate diagnosis. These diagnostic values are the first of their kind in ergonomics; hence, making comparative statements is not possible. Moreover, this model outperforms dental specialists and can provide real-time feedback. In the present study, the reliability of human raters was very low, which may be attributed to the subjectivity involved in interpreting posture among them. Hence, this AI model can serve as a valuable tool to assist dental professionals in making better decisions regarding ergonomic working postures.

Our AI model indicates a promising potential for improving diagnostic accuracy, particularly by helping dentists adopt ergonomic working postures. Our model represents a collaborative effort between YOLOv11 and MediaPipe, as each framework addresses unique requirements. Consequently, we consider this collaboration to enhance the accuracy of pose estimation and the efficiency of real-time performance. The automated identification of anatomical landmarks from images, along with the recognition of critical points such as the shoulders, hips, knees, and head, enables these models to calculate angles and both vertical and horizontal alignments that assess various aspects of posture changes. This technology eliminates the need for manual measurements and subjective evaluations, promoting a more standardized and data-driven approach to Posture Assessment [8,23]. The validity of MediaPipe has been thoroughly investigated. Lafayette et al. [42] conducted a quantitative and statistical evaluation of MediaPipe’s angular estimation capabilities, demonstrating that it exhibits exceptional absolute error rates when compared to clinical standards. Additionally, its validity is further supported by achieving 100% accuracy in classifying exercise postures and detecting human movements in non-standard videos, with an overall accuracy exceeding 90% [43].

Ergonomic evaluations in the field of dentistry offer numerous benefits, particularly in reducing MSDs. These assessments help identify and address risk factors in dental practices that contribute to MSDs, such as inappropriate postures, repetitive movements, and excessive physical strain. Additionally, improvements in ergonomics can enhance job satisfaction and efficiency, leading to reduced fatigue and improved mental well-being [44,45]. Dentists may encounter challenges in decision-making within this field due to constraints such as limited time and inadequate training on assessment criteria. Thus, this AI model can serve as a valuable guide, empowering them to make better decisions and improve their performance. The tested AI model can serve as a self-assessment tool that can assist dental professionals in adopting and implementing proper work postures, which can help reduce the risk of MSDs.

Several studies have reported that these MSDs often begin at the university level during students’ education [46,47,48]. Additionally, while the risks associated with MSDs are taught to and understood by students during the early stages of their training, this occupational health concern tends to receive less attention as time progresses [49]. Hence, considering the ergonomic risks in dentistry, it is crucial to implement effective ergonomic interventions to prevent and address these issues. An important initial step in mitigating risks and enhancing worker protection is identifying ergonomic risk factors in the workplace. Data on the ergonomic postural assessment of dental professionals from previous research are extremely limited. Therefore, recognizing ergonomic risk factors in the workplace is an essential first step in correcting hazards and improving worker safety. Our AI model can be highly effective in identifying ergonomic risk factors when implemented in dental schools. A real-time feedback system on the ergonomic postures adopted by dental students can emphasize the importance of adhering to the principles of dental ergonomics early in their careers, helping to prevent the development of MSDs among them.

It is crucial to understand that our measurement system exclusively classifies ten predefined postures. Our approach has effectively enabled us to identify the classification algorithm and parameters that yield the best results for the known sitting posture, which exhibits the most similar input channel pattern. In the present study, we utilized a smaller number of datasets. To address this limitation, we employed transfer learning by fine-tuning YOLOv11 (pre-trained on the large COCO dataset) and MediaPipe (trained on diverse pose datasets). Transfer learning enabled us to leverage rich feature representations learned from millions of images, thereby enhancing model robustness and reducing overfitting despite the smaller dataset size. Additionally, we applied data augmentation techniques, including flipping, scaling, and rotation, to increase variability and improve generalizability. Our results demonstrated that this method achieved the highest performance. This automated model can assist in the detection of even minor postural deviations, which can serve as early indicators of emerging musculoskeletal disorders. This capability facilitates the implementation of timely preventive measures. While the current study demonstrates the methodological validity of our model, its eventual translation into clinical settings requires careful consideration of both hardware and software feasibility. Importantly, one strength of this approach is that it does not require highly specialized imaging equipment; instead, it can be implemented using standard devices commonly found in many practices. For example, a modern smartphone or tablet equipped with a high-resolution camera, such as those available on contemporary iOS or Android devices, would be sufficient for image acquisition. In clinics with established imaging infrastructure, integration with existing intraoral or chairside cameras could further enhance usability without incurring additional capital expenditure.

In the present study, we encountered a few limitations with 2D imaging, particularly its inability to assess rotation between planes. Another limitation was the number of datasets, which were obtained from a single institution. These factors may restrict the generalizability of our findings across diverse clinical settings. Future research should involve larger, multi-center datasets, the inclusion of video data, and 3D posture analysis to further validate and extend our results. Despite these limitations, the use of transfer learning and data augmentation significantly improved robustness, and our findings demonstrate that AI-based assessment can serve as a valuable tool for evaluating working postures.

### Implications and Future Research

The current model was developed based on criteria focusing on sitting posture; however, in the future, our model will be further trained and tested for ergonomics related to various postures, including standing posture. Future research directions should also incorporate video analysis to evaluate the work postures of dentists. Additionally, it is highly valuable to explore multi-person posture recognition to identify the work postures of dental assistants as well. Future studies should consider three-dimensional imaging to address the limitations of two-dimensional imaging, which lacks depth perception, by providing a comprehensive spatial understanding of movement and posture. This capability enables more precise calculations of joint angles and improved risk assessments for MSDs during ergonomic evaluations. In contrast, traditional two-dimensional methods often fail to accurately capture the full three-dimensional movement of joints and may misrepresent posture. Three-dimensional systems, such as motion capture technology, offer a more extensive dataset that allows for accurate angle calculations and the identification of risk factors, including inappropriate postures and repetitive movements. Furthermore, maintaining ethical integrity is of utmost importance when using AI in health assessments for professionals. Therefore, obtaining valid informed consent that explicitly addresses the confidentiality of data usage, storage, and assessment is essential. Participants must be informed about the use of AI in their assessment, understand its limitations, and have the right to consent to its application in their care.

## 5. Conclusions

Ergonomic evaluations in the field of dentistry provide numerous benefits, particularly in reducing MSDs. This AI model has demonstrated exceptional performance in assessing the working postures of dental professionals. Our model outperformed human evaluators, establishing this AI-based model as a reliable tool for Posture Assessment. It has the potential to serve as the ‘gold standard’ in evaluating the ergonomics of dental operators. To achieve external validation of this model, further research will be conducted using larger datasets obtained through collaboration with other institutions in the region. This automated model can detect even minor postural deviations, which may serve as early indicators of developing musculoskeletal disorders. This capability enables self-assessment and the adoption of timely preventive measures. This AI model can serve as a reliable and straightforward method for evaluating the working postures adopted by dental professionals while treating patients, as well as for assessing dental students during their training in dental schools.

## Figures and Tables

**Table 1:** Modified-Dental Operator Posture Assessment Criteria (M-DOPAC).

Posture Aspects	Scoring Criteria		
	Acceptable (1 Point)	Compromised (2 Points)	Harmful (3 Point)
hips	Level on stool	Hips not level on stool	Not applicable
trunk	Front to back ≤ 20°	20° < Front to back < 45°	Front to back ≥ 45°
	Side to side ≤ 20°	20° < Side to side < 45°	Side to side ≥ 45°
head/neck	Front to back ≤ 20°	20° < Front to back < 45°	Front to back ≥ 45°
	Side to side ≤ 20°	20° < Side to side < 45°	Side to side ≥ 45°
shoulders	Relaxed	Slummed forward	Not applicable
	Both shoulders level with trunk	One or both shoulders elevated above line of trunk	Not applicable
upper arms	Upper arms parallel to long axis of torso	<20° abduction away from body	>20° abduction away from body
	Elbows at waist level	Elbows at waist level but <60°	Elbows at waist level but >60°
wrist	Flexion or extension of either wrist ≤ 15°	Flexion or extension of either wrist > 15°	Not applicable

**Table 2:** Cohen’s Kappa statistics of calibrated evaluators, AI-based model, and each of the five sets scored by human evaluators against the AI, along with Intra Class Correlation and Intra Rater Reliability.

Cohen’s Kappa Statistics for Inter-Rater Reliability of Test Set Evaluated by Calibrated Principal Evaluators						
Evaluator	Total Sets Evaluated		Kappa Value			Sig
Principal Evaluators	50		0.924			0.000 *
**Cohen’s Kappa statistics for inter-rater reliability of 50 sets scored by an AI model against the scores generated by calibrated principal evaluators**						
**Evaluator**	**Total Sets Evaluated**		**Kappa Value**			**Sig**
AI-based model	50		0.922			0.000 *
**Cohen’s Kappa Statistics for Each of the Ten Sets Scored by Human Evaluators Against the Scores Generated by the AI Model**						
**Evaluator**	**Total Sets Evaluated**		**Kappa Value**			**Sig**
Evaluator 1	10		−0.071			0.598
Evaluator 2	10		0.000			1.000
Evaluator 3	10		−0.053			0.725
Evaluator 4	10		−0.053			0.725
Evaluator 5	10		0.429			0.050
**Intra Class Correlation (ICC)-Intra Rater Reliability Assessment (IRR)**						
**Evaluators** **(1st vs. 2nd evaluation)**	**ICC-Single Measures**	**ICC** **Averaged Measures**	**F Test Value**	***p*** **Value**	**Average Measures**	
					**Lower 95%** **CI**	**Upper 95%** **CI**
Evaluator 1	−0.286	−0.800	0.600	0.771	−0.897	0.440
Evaluator 2	0.053	0.100	1.111	0.439	−0.563	0.634
Evaluator 3	0.050	0.092	1.111	0.439	−2.580	0.776
Evaluator 4	0.000	0.000	1.000	0.500	−4.189	0.765
Evaluator 5	0.426	0.597	2.379	0.106	−0.740	0.902
AI	1.000	1.000	25.000	0.000 *	-	-

* Significant.

**Table 3:** Crosstabulation to assess sensitivity and specificity for AI-based diagnosis with that of calibrated evaluators.

Calibrated Evaluators and AI Model Diagnosis of Operator Ergonomics					
			AI-Based Diagnosis		Total
			Positive	Negative	
Calibrated Evaluators	Positive	Count	42	1	43
		% within Calibrated Evaluators	97.7%	2.3%	100.0%
		% within AI Scores	97.7%	14.3%	86.0%
	Negative	Count	1	6	7
		% within Calibrated Evaluators	14.3%	85.7%	100.0%
		% within AI Scores	2.3%	85.7%	14.0%
Total		Count	43	7	50
		% within Calibrated Evaluators	86.0%	14.0%	100.0%
		% within AI Scores	100.0%	100.0%	100.0%
**Human Evaluation and AI-Based Diagnosis of Operator Ergonomics**					
			**AI-Based Diagnosis**		**Total**
			**Positive**	**Negative**	
Human Evaluation	Positive	Count	8	10	18
		% within Human Evaluation	44.4%	55.6%	100.0%
		% within AI-based diagnosis	20.5%	90.9%	36.0%
	Negative	Count	31	1	32
		% within Human Evaluation	96.9%	3.1%	100.0%
		% within AI-based diagnosis	79.5%	9.1%	64.0%
Total		Count	39	11	50
		% within Human Evaluation	78.0%	22.0%	100.0%
		% within AI-based diagnosis	100.0%	100.0%	100.0%

**Table 4:** Positive predictive value (PPV) and negative predictive value (NPV) based on AI-based model diagnosis.

Parameter	B	Std. Error	95% Profile Likelihood Confidence Interval				Hypothesis Test			
			Lower		Upper		Chi-Square		df	Sig.
(PPV)	0.977	0.023	0.902		0.999		180.60		1	0.000 *
(NPV)	0.857	0.132	0.506		0.991		42.000		1	0.000 *
**Specificity**										
**Parameter**	**B**	**Std. Error**	**95% Profile Likelihood Confidence Interval**				**Hypothesis Test**			
			**Lower**		**Upper**		**Chi-Square**		**df**	**Sig.**
(Intercept)	0.857	0.132	0.506		0.991		42.000		1	0.000 *
**Sensitivity**										
**Parameter**	**B**	**Std. Error**	**95% Profile Likelihood Confidence Interval**				**Hypothesis Test**			
			**Lower**		**Upper**		**Chi-Square**		**df**	**Sig.**
(Intercept)	0.977	0.023	0.902		0.999		180.60		1	0.000 *
**Risk Estimate**										
				**Value**		**95% Confidence Interval**				
						**Lower**		**Upper**		
Odds Ratio for Predicted (1/2)				252.000		13.855		458.353		
For Cohort Actual = 1				6.837		1.113		41.995		
For Cohort Actual = 2				0.027		0.004		0.193		
N of Valid Cases				50						

Significance (Sig)—* *p* < 0.05.

**Table 5:** Positive predictive value (PPV) and negative predictive value (NPV) based on human evaluation.

Parameter	B	Std. Error	95% Profile Likelihood Confidence Interval					Hypothesis Test			
			Lower			Upper		Chi-Square		df	Sig.
(PPV)	0.444	0.117	0.234			0.670		14.400		1	0.000 *
(NPV)	0.031	0.308	0.002			0.130		1.032		1	0.310
**Specificity**											
**Parameter**	**B**	**Std. Error**		**95% Profile Likelihood Confidence Interval**				**Hypothesis Test**			
				**Lower**		**Upper**		**Chi-Square**		**df**	**Sig.**
(Intercept)	0.091	0.086		0.005		0.343		1.100		1	0.294
**Sensitivity**											
**Parameter**	**B**	**Std. Error**		**95% Profile Likelihood Confidence Interval**				**Hypothesis Test**			
				**Lower**		**Upper**		**Chi-Square**		**df**	**Sig.**
(Intercept)	0.205	0.064		0.099		0.348		10.065		1	0.002 *
**Risk Estimate**											
					**Value**		**95% Confidence Interval**				
							**Lower**		**Upper**		
Odds Ratio for Predicted (1/2)					0.026		0.003		0.232		
For cohort Actual = 1					0.226		0.118		0.430		
For cohort Actual = 2					8.744		1.340		57.046		
N of Valid Cases					50						

Significance (Sig)—* *p* < 0.05.

**Table 6:** Area Under the Curve (AUC) based on human evaluation and AI model for Dental Operator Ergonomics Evaluation.

Summary of Outcomes in AI-Based Evaluation				
Outcome			N	
Positive			43	
Negative			07	
**Summary of Outcomes in Human Evaluation**				
**Outcome**			**N**	
Positive			32	
Negative			18	
**Area Under the Curve**				
**Test Result Variable(s): AI**				
**AI-based Diagnosis Area Under the Curve**	**Std. Error ^a^**	**Asymptotic Sig. ^b^**	**Asymptotic 95% Confidence Interval**	
			**Lower Bound**	**Upper Bound**
0.917	0.079	0.000	0.762	1.000
**Test Result Variable(s): Human Evaluation Score**				
**Human Evaluation Area Under the Curve**	**Std. Error ^a^**	**Asymptotic Sig. ^b^**	**Asymptotic 95% Confidence Interval**	
			**Lower Bound**	**Upper Bound**
0.143	0.059	0.000	0.027	0.259

Larger values of the test result variable indicate stronger evidence for a positive actual state. ^a^ Under the nonparametric assumption. ^b^ Null hypothesis: true area = 0.5.

## Data Availability

The original contributions presented in this study are included in the article. Further inquiries can be directed to the corresponding author.

## Abbreviations

Conv	Convolutional Block
C3k2	Cross-stage partial bottleneck with 3 convolution layers and kernel size 2
Concat	Concatenation of feature maps from different scales.
Upsample	Bilinear (sometimes nearest-neighbor) upsampling to double feature map resolution.
SPPF/SPFF	Spatial Pyramid Pooling (Fast) captures multi-scale receptive fields by pooling at different kernel sizes.
C2PSA	Cross-Stage Partial with Partial Self-Attention (lightweight attention to refine features).
